# Maintaining semen quality by improving cold chain equipment used in cattle artificial insemination

**DOI:** 10.1038/srep28108

**Published:** 2016-06-17

**Authors:** Daniel Lieberman, Elizabeth McClure, Stephen Harston, Damian Madan

**Affiliations:** 1 Intellectual Ventures Laboratory, Bellevue, Washington, United States of America

## Abstract

Artificial insemination of dairy cattle is a common practice in the developing world that can improve farmer incomes and food security. Maintaining the fertilizing potential of frozen semen as it is manipulated, transported and stored is crucial to the success of this process. Here we describe simple technological improvements to protect semen from inadvertent thermal fluctuations that occur when users mishandle semen using standard equipment. We show that when frozen semen is mishandled, characteristics of semen biology associated with fertility are negatively affected. We describe several design modifications and results from thermal performance tests of several improved prototypes. Finally, we compare semen that has been mishandled in standard and improved equipment. The data suggest that our canister improvements can better maintain characteristics of semen biology that correlate with fertility when it is mishandled.

Over the last few centuries selective breeding has developed dairy cattle breeds that produce large quantities of milk. By contrast, indigenous cows in the developing world are far less productive^1^,^2^. Recognizing this discrepancy, many developing countries strive to improve milk yields—and therefore farmers’ incomes and food security—by utilizing the genetics of nonendemic breeds. While traditional mating of imported cattle is used to generate both cross and full breeds, artificial insemination (AI) is the prevailing method used to accomplish this goal^3^.

Perhaps the greatest breakthrough to impact AI came in the 1950s when glycerol was discovered to act as a cryoprotectant for dairy bull semen^4^,^5^. This advance allowed for a greater temporal, and therefore spatial, separation between semen collection and insemination. Now, semen is generally collected, packaged into semen straws, and frozen at centralized facilities. The frozen semen is then shipped throughout the world in Dewars containing liquid nitrogen (LN) (Fig. 1a).

Four variables affect whether AI will result in conception: heat detection, inseminator efficiency, fertility of the cow, and the fertility of the semen^6^,^7^. Mammals are fertile for a limited window of time during each reproductive cycle, so effectively identifying when a cow is in heat is vital for AI success. Similarly, the competency of an inseminator is important to properly deliver semen hygienically and on target. These first two variables are typically managed through proper AI technician training. Cow fertility, the third variable influencing AI success, is affected by diseases such as mastitis, the length of the post-partum waiting period, and nutrition, and is influenced by managing the cow throughout its life.

Semen fertility, the final determinant of AI success, is most commonly affected by handling subsequent to semen packaging and freezing^8^. A vast amount of research has optimized the number of sperm packed per straw, the additives that supplement semen, and the precise protocols for properly freezing this mixture^9^,^10^,^11^. Once semen is frozen into individual straws, most bull stud ranches perform quality control assessments by assaying *in vitro* characteristics that are associated with fertilizing potential^12^,^13^. Consequently, semen sold by most reputable bull stud ranches is highly fertile, and if stored properly in LN, will retain its fertilizing potential indefinitely^6^.

However, improper handling by prematurely breaking the cold chain can dramatically decrease semen fertility (Fig. 1). In order to prevent this thermally-induced semen damage, most developed world bovine AI technicians are taught to remove semen straws from LN for only very brief periods, unless the straw is being thawed for immediate usage. Training procedures limit exposures to eight, five or even three seconds^14^. These rules are stressed during technician training because very short exposures to ambient temperatures can cause large temperature fluctuations within the straws^15^. While these fluctuations are often not large enough to thaw the straws’ contents, they do cause cumulative and irreversible damage that negatively impacts the fertilizing potential of semen^10^,^14^,^16^,^17^,^18^.

Some of the equipment used to store and categorize straws in a Dewar was designed to promote proper handling. The plastic goblets and cane assemblies used in large Dewars help improve protection from inadvertent exposure^15^. Large Dewars, however, are used less frequently in developing countries where farms are typically smaller, increasing the need for AI technicians to be mobile. In these settings 3 L or smaller Dewars are most common as they are easily transported by motorcycle^19^. The smaller size of these Dewars does not allow the use of the canes. Consequently, goblets are less common and straws are often placed directly in the canister.

A limited number of studies and a large amount of anecdotal evidence suggest that poor handling of frozen bovine semen is a frequent occurrence. One study compared several morphological and motility characteristics in semen stored at operational farms and a facility where proper handling practices were tightly controlled. Semen stored at the farm showed significant damage, suggesting that proper handling guidelines were not followed^20^. A more recent study measured *in vitro* fertilization rates of semen stored at a variety of locations. The authors indicate that when poor quality semen straws—attributed largely to routine poor handling practices—were excluded from the procedure, embryo fertilization improved 9%^14^,^21^.

Training is often less rigorous in the developing world, implying that handling-induced semen damage is likely a greater problem in these areas. Indeed, the number of AI services per conception is higher in developing countries^3^, and improper semen handling contributes to these differences^22^,^23^,^24^.

In an effort to improve AI success rates in the developing world, we sought to develop inexpensive and easy to use technology that protects bovine semen from thermal fluctuations. First, we quantified aspects of semen damage that occur when frozen straws are subjected to common poor handling practices. Then, we designed and tested the thermal performance of three improved semen canister prototypes. Finally, we subjected frozen semen stored in a standard and improved canister to repeated ambient temperature exposures. Subsequent analysis showed that semen stored in the improved canister, which limited thermal fluctuations, possessed characteristics of more highly fertile semen.

## Results

### Quantifying Semen Damage

**Figure 1:**
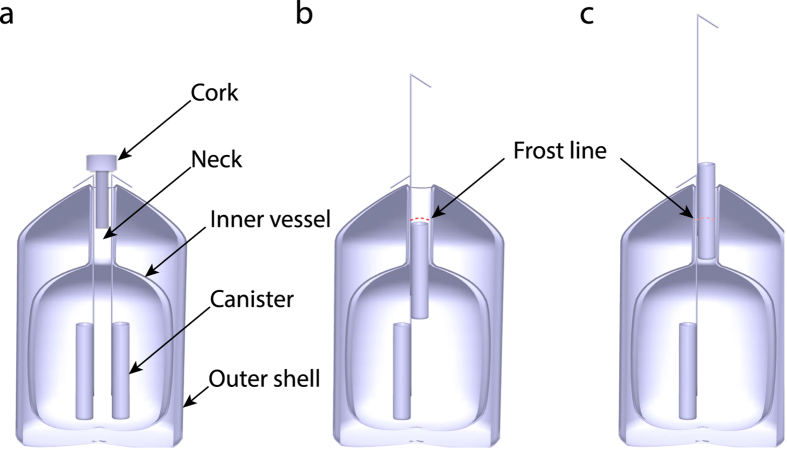
Depiction of poor handling practices. The anatomy of a LN Dewar with the canisters (**a**) in the storage position, (**b**) using recommended practices to access a canister, and (**c**) using common poor handling practices are shown. Note that the top of the canister is below the frost line in **b** and above the frost line in **c**.

Generally, Dewar canisters contain multiple semen straws. If an AI technician uses poor handling practices then each time he or she removes a straw for use, the remaining straws within the inventory will experience multiple thermal exposures (Fig. 1). Similar events could occur during the transfer of straws between Dewars, such as when importers transfer straws to distribution centers, distributers transfer straws to AI technicians, etc.

**Figure 2:**
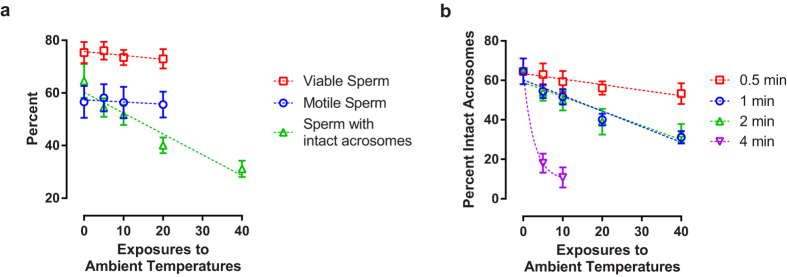
Poor handling damages semen. (**a**) Frozen semen straws were subjected to repeated one minute ambient temperature exposures. Measurements of membrane viability staining, sperm motility, and acrosome integrity are displayed. (**b**) Frozen semen was subjected to multiple ambient temperature exposures at 0.5, 1, 2, and 4 minute durations. The post-thaw acrosome integrity measurements are shown. For both graphs, the fitted lines are included as visual guides (n = 6, error bars represent standard deviation (SD)).

To quantify the damage associated with repeated thermal exposures, we subjected the canister of a standard 3 L Dewar containing frozen bovine semen to recurrent ambient temperature exposures. We repeated these cycling treatments at various exposure durations, and between each exposure the canister was submerged in LN. After cycling treatments were complete, we measured aspects of semen biology that correlate with fertility^12^,^13^. Both semen motility and viability measurements were resistant to these treatments (Fig. 2a; motility: *F*_1,22_ = 0.29, *P* = 0.49, viability: *F*_1,22_ = 2.39, *P* = 0.13). Surprisingly, 20 one-minute ambient exposures were not sufficient to induce measureable damage to these physiological characteristics.

However, damage to the acrosome was pronounced (Fig. 2a; *F*_1,28_ = 85.48, *P* < 0.00001). The acrosome is a large vesicle located at the anterior region of the sperm head that contains hydrolytic enzymes and surface antigens necessary for the acrosome reaction, a necessary process in fertilization where the sperm penetrates the zona pellucida of the egg^25^. Measurements showed that under all tested durations of exposure, acrosome damage increased with the quantity of exposures (Fig. 2b; 0.5 min exposure: *F*_1,28_ = 17.64, *P* = 0.0002; 1 min exposure: *F*_1,28_ = 144.90, *P* < 0.00001; 2 min exposure: *F*_1,28_ = 73.47, *P* < 0.00001; 4 min exposure: *F*_1,16_ = 69.60, *P* < 0.00001). These data are consistent with reports that show that acrosomes are dynamic structures particularly sensitive to damage^14^,^26^.

### Canister Improvement Design

**Figure 3:**
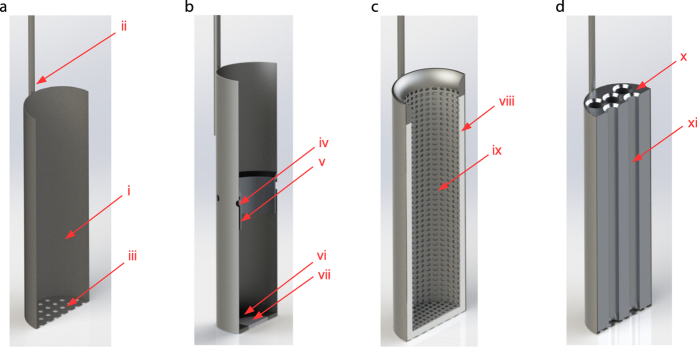
Prototype schematics. (**a**) A depiction of a canister found in many small portable Dewars is shown. The cylindrical cup (i) that holds semen straws it attached to a handle (ii) that allows manipulation inside a Dewar and to rest on a hook at the neck. A grate (iii) or drain holes are incorporated into the canister base to allow LN to empty as it is raised. (**b**) Prototype 1 is shown. When the canister is in the resting position in the Dewar, LN enters the canister through drain holes (iv). When the canister is raised cold nitrogen vapor fills the top half of the canister as LN maintained at the base of the canister boils. A vapor guard (v) is included to minimize vapor exit via the drain holes. The grate at the base of the canister is replaced with a solid metal surface (vi) to prevent LN draining. To accommodate low levels of LN in a Dewar, the canister is fitted with sintered metal that acts as a permeable membrane to allow LN to slowly fill over several hours (vii). (**c**) Prototype 2 is shown. This canister is lined with an LN absorbent material (viii) to both provide thermal insulation from the environment and blanket semen straws with cold nitrogen vapor as the liquid vaporizes. A grated metal surface (ix) is included to protect the LN absorbent material. (**d**) Prototype 3 is shown. A solid aluminum cylinder (x) is designed to fit snugly within the canister to increase thermal mass. A system of holes (xi) is drilled along its length to accommodate semen straws.

The canisters found in most small portable Dewars used for AI consist of a cylindrical cup with a handle to allow manipulation inside a Dewar and to rest on a hook at the neck. A grate or drain holes are incorporated to the canister base to allow LN to flow out as it is raised (Fig. 3a).

We conceived of three approaches to improve the canister design to decrease thermal fluctuations within semen straws when the canister is removed from the Dewar.

In the first approach (Prototype 1) we sought to maintain a level of LN within the canister by replacing the grate at the canister base with a solid metal surface (Fig. 3b). We designed a canister that possesses drain holes drilled along the circumference of the canister approximately half way (5 cm) from its bottom to allow LN to flow between the canister and the Dewar. In addition, the drain holes allow the canister to sink when it is inserted into the Dewar. This prototype is similar to several existing canisters with the notable difference that our design allows for significantly higher levels of LN in the canister.

The intention of Prototype 1 was to drive cold nitrogen vapor over the top portion of the straws that are not submerged in LN. However, when we removed our canister prototype from the Dewar neck, we noticed that a significant portion of nitrogen vapor escapes from the drain holes. To minimize this effect we modified the design to include a vapor guard on the inside wall of the canister along the circumference that covers the drain holes and extends below the LN liquid level (Fig. 3b).

To accommodate low levels of LN in a Dewar we fitted the canister with sintered metal that acts as a permeable membrane to allow LN to slowly fill over several hours while restricting the loss of LN during a straw extraction event lasting up to a few minutes (Fig. 3b). The use of this prototype extends the effectiveness of this device when the LN level in the Dewar drops below the elevation of the drain holes.

In the second approach (Prototype 2) we sought to provide thermal insulation from the environment and blanket semen straws with cold nitrogen vapor as LN vaporizes. To accomplish this goal we designed a canister lined with an LN absorbent material (Fig. 3c). The thickness of the material is intended to store sufficient LN for an exposure lasting several minutes. We also included the LN absorbent material at the canister base to allow liquid to fill and drain during use but minimize natural convection of cold vapors when the canister was raised in the Dewar.

In the final approach (Prototype 3) we sought to increase thermal mass of the canister to slow the temperature rise during an exposure (Fig. 3d). We machined a solid aluminum cylinder to fit snugly within the canister and drilled a system of holes along its length to accommodate several semen straws. This prototype increases organization within the canister allowing straws from different bulls to be separated in a canister similar to how goblets function in larger Dewars.

### Thermal Performance

We subjected the prototypes to ambient exposure tests by placing thermocouple temperature sensors at the top, middle, and bottom of a semen straw. Each prototype contained three straws with thermocouples. We raised the canister prototypes completely out of a LN-filled Dewar for approximately one minute then re-submerged into the Dewar. The experiment was performed in a laboratory held at 23 °C ± 0.2 °C throughout the experiment.

**Figure 4:**
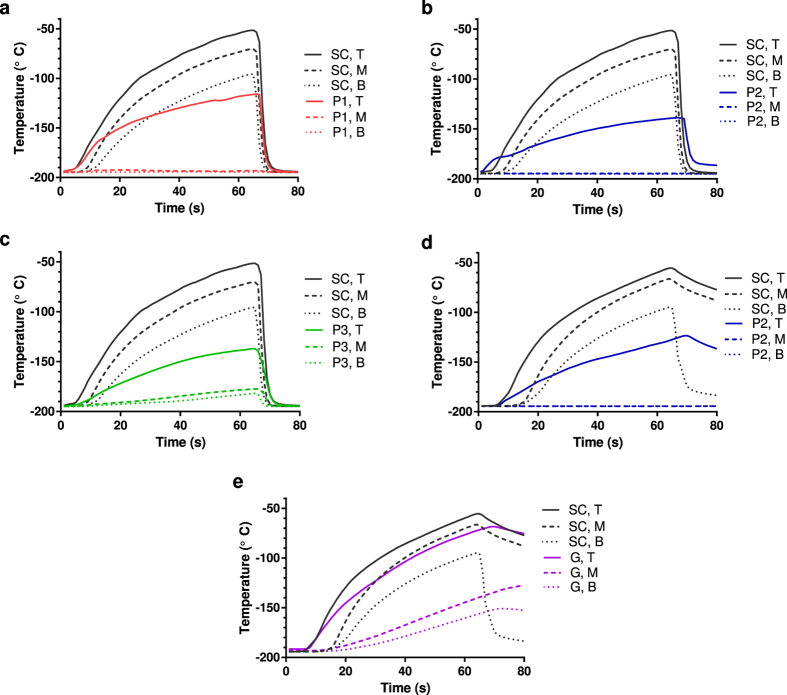
Prototypes reduce thermal fluctuations within semen straws during poor handling. Temperature measurements at the top (T), middle (M), and bottom (B) of a semen straw placed within Prototype (P) 1, 2 and 3 (subfigs. (**a–c**) respectively) removed from a full 3 L Dewar for approximately one minute then re-submerged into LN are shown. Readings from similar experiments using a standard canister (SC) are shown for reference. (**d**). The experiment in subfig. b was repeated with a Dewar that was 25% full of LN. (**e**) The experiment in subfig. d was repeated with a standard canister containing straws held within an empty plastic goblet (G). All plots report values as mean readings from thermocouples located on three different semen straws from the same ambient exposure (n = 3). Data are representative of three independent experiments.

All three prototypes demonstrated an improvement in delaying a temperature rise in the straws (Fig. 4). The three prototypes effectively maintained the mid and lower portion of the straw near the −196 °C LN temperature. Prototypes 2 and 3 were slightly more effective at retaining the temperature in the top portion of the straw. Thermal response simulations of the standard canister using a multiphysics modeling and simulation tool (COMSOL, Stockholm, Sweden) suggest natural convection is a driving factor and likely marginalizes potential forced convection effects in this experiment when the canister is raised (Supplementary Fig. 1). The effect of convection from small oscillations while holding the canister and air currents in the lab were not simulated but are likely small compared to convection from raising the canister.

**Figure 5:**
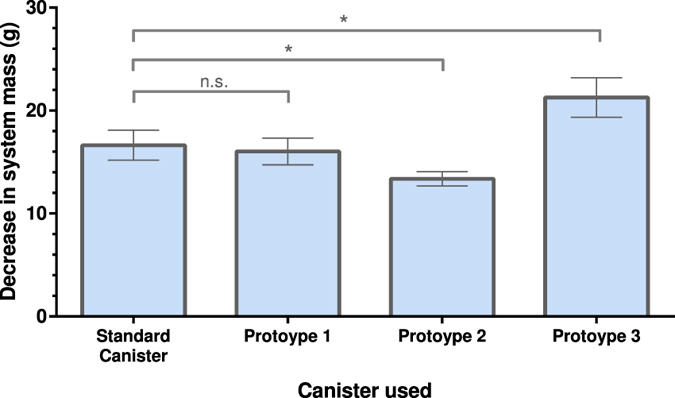
Comparison of LN loss after poor handling. Canisters were removed from a full 3 L Dewar for one minute then reintroduced to the system. The loss in mass is shown (n = 3, error bars represent SD). Data were analyzed with an ANOVA followed by pairwise comparisons of each prototype with the standard canister (* denotes p ≤ 0.05, n.s. denotes not significant).

To compare LN loss due to cycling the canisters, we measured the change in mass of the entire system, *i.e*. the Dewar and canister (Fig. 5). The different types of canisters significantly affected the change in mass due to loss of LN (*F*_3,8_ = 16.27, *P* = 0.0009). The standard canister results in the loss of approximately 16 g of LN. Pairwise comparisons of each prototype revealed that Prototype 1 resulted in a similar loss of LN (*P* = 0.91). Prototype 2 lost approximately 13 g, a slight improvement on the standard canister (*P* = 0.05), while Prototype 3 consumed more LN at 21.3 g (*P* = 0.009). We note that the LN mass loss for a given prototype was a combination of LN that evaporated when the canister was exposed to the environment and from the boiling that occurred when the canister was re-inserted into the Dewar.

### Protection of Semen from Damgage

**Figure 6:**
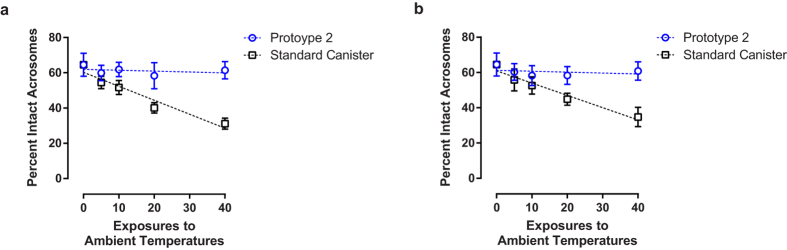
Prototype 2 protects semen from poor-handling-induced damage. Frozen semen straws were placed within either a standard canister or Prototype 2 in a full (**a**) or 25% full (**b**) Dewar and exposed to up to 40 one minute ambient temperature exposures. The post thaw acrosome integrity measurements are shown (n = 6, error bars represent SD). Fitted lines are included as visual guides.

Since Prototype 2 demonstrated improvement in maintaining temperature and reducing the loss of LN, we tested its ability to protect against semen damage in comparison to the standard canister. Prototype 2 was subjected to the same ambient temperature exposure regimen as previous described for the standard canister (1 minute exposures for up to 40 exposures). As opposed to the standard canister, which was previously demonstrated to lead to a decrease in acrosome integrity (Fig. 2), semen stored in a Prototype 2 canister showed no increase in damage with increasing exposures, as measured by post-thaw acrosome integrity measurements (Fig. 6a; *F*_1,28_ = 0.46, *P* = 0.50). We conducted these same trials but when the Dewar was only 25% full of LN, and achieved similar results (Fig. 6b; standard canister, *F*_1,28_ = 92.3, *P* < 0.00001; prototype 2 canister, *F*_1,28_ = 0.50, *P* < 0.49). These data strongly suggest that a canister lined with an LN absorbent material can better maintain characteristics of semen biology that correlate with fertility when subjected to repeated poor handling.

## Discussion

Here we show that damage associated with improper access of cryogenically stored bovine semen is mitigated with simple and inexpensive improvements to the storage system. An alternative solution to this problem is to train AI technicians and semen handlers to avoid exposing frozen semen straws to non-LN temperatures for more than a few seconds. While proper technique is stressed in many training programs, the problem of exposure-induced damage persists and is suspected to be more severe in the developing world.

It is likely that the majority of semen damage caused during improper frozen semen access is inadvertent. Inadequately trained users might assume that exposing frozen semen to ambient conditions is not damaging as long as the contents remains in a frozen state. However, thermal damage can occur after very brief ambient exposures that are not sufficient to thaw the semen.

Studies suggest that damage due to brief ambient exposures results when the bulk product temperature rises above −137 °C^27^—the glass transition temperature of water—and then cools back down when re-introduced to LN^14^,^28^. The reordering of water molecules during these events likely affects multiple aspects of semen biology associated with fertilizing potential. While these insults are presumably stochastics, they appear to have a greater chance of negatively affecting the integrity of biological membranes since we observed damage via the acrosomal assay but not the motility or viability assays. The exact mechanism of this phenomenon has yet to be fully elucidated, but it may share similarities to cryocapacitation, a form of cryoinjury that can occur during the freezing process that largely affects sperm outer and acrosomal membranes^26^,^29^.

In this study we exposed frozen bovine semen to poor practices that mimic improper access and subsequently measured semen damage. The relationship between exposure time and acrosome damage is complex but our data are consistent with the hypothesis that damage accumulates upon fluctuations about the glass and freezing transitions. To decrease thermal fluctuations within the canister contents, we designed several simple and inexpensive improvements to the Dewar canister. These improvements, such as canisters lined with an LN absorbent material, better maintain characteristics of semen that are associated with fertilizing potential.

We hesitate to extrapolate the effect that this technological mitigation would have on actual fertilization rates. Poor handling is one of several factors that affect AI success and, poor handling behaviors occur with variable frequencies and severities, making it difficult to quantify their full effect. Furthermore, we constrained our evaluation to a select number of aspects of sperm biology that are necessary for fertilization^25^. Of the biological aspects we investigated, we suspect that the assays do not fully reflect damage that poor handling imparts. For example, the results from the acrosomal integrity assay are reliant on gross morphological differences in these structures. We suspect that this assay underestimates the level of acrosomal damage by failing to register submicroscopic injuries that negatively affect fertility.

Since all current *in vitro* semen assays suffer from some degree of sensitivity limitations and because each assay measures only a subset of aspects needed for a semen straw to result in cow fertilization—including *in vitro* fertilization^30^— predicting the fertilizing potential of a semen straw in a laboratory setting is a dubious exercise^12^,^13^,^31^. Therefore, while encouraging, we are reluctant to extrapolate the impact of our Dewar improvements without a full-fledged field study.

The intention of this study was to find technological solutions that would lead to higher AI success rates in the developing world. Of course, any technological solution will need to pass several hurdles, in addition to such a technical evaluation, for it to achieve this goal. Among these challenges are those associated with any requirements on the user to interact with the equipment differently than they currently do.

In a field observation in Kenya we sought feedback regarding prototypes based on all three prototypes. All observations and interviews with AI professionals found no significant differences in how the prototype canisters were handled or managed as compared to standard equipment (Supplementary Fig. 2).

Among the learnings from this evaluation was the observation that, in an effort save on LN costs, AI technicians in rural Kenya invariably operate with partially filled LN Dewars. To ensure that the performance of the prototypes mentioned in this report do not suffer as a result of this behavior, we repeated thermal performance and acrosomal integrity assays with partially filled Dewars. Results from these studies suggest that the protection that the Prototype 2 canister imparts is minimally affected by the amount of LN in the Dewar (Figs 4d and 6b).

Plastic goblets represent an alternative means to protect semen straws from poor handling. These containers fill with LN as long as the LN level in the Dewar is higher than the lip of the goblet. When the level of LN is insufficiently high to fill a goblet, such as in a 25% full 3L Dewar, the empty goblet does maintain lower straw temperatures during poor handling as compared to a standard canister. However, under these conditions, Prototype 2 demonstrates a significantly improved ability to maintain the semen at lower temperatures (Fig. 4d,e, Supplementary Table 1).

Our findings are applicable to industries outside of the AI industry. Maintenance of the cold chain is crucial to preserve the integrity of a variety of biological samples such as seeds, oocytes, blood products, embryos, stem cells, and tissues from humans, animals and plants^32^. Inventory control methods such as those described here could limit thermal fluctuations within these samples and protect them from cyrodamage. These technologies may be especially useful for samples frozen using vitrification, a process often used in the preservation of oocytes, embryos, and stem cells, which leaves the samples especially sensitive to devitrification damage^33^.

## Materials and Methods

### Reagents

Unless otherwise indicated, reagents used in this study were purchased from Sigma (St. Louise, MO). All frozen semen used in the study was purchased from Accelerated Genetics (Baraboo, WI). The semen originated from a single lot from the Holstein Bull Michigan Frost (014HO07313) and was packaged in 0.5 ml French straws.

### Motility and Membrane Viability Staining

Frozen semen straws were thawed in a water bath set at 35 °C for 30 seconds. Motility and membrane viability staining were performed using computer assisted semen analysis (CASA). Briefly, for motility staining 20 μL of semen was gently mixed with 30 μL Easybuffer B (IMV Technologies, Maple Grove, MN) and 50 μL of 1 mg/ml Hoechst 33342. Samples were incubated at 35 °C for 20 minutes, loaded onto pre-warmed four chamber slides (Leja, Nieuw-Vennep, The Netherlands) and imaged using the Animal Motility package on an IVOS II (Hamilton Thorne, Beverly, MA) system using version 1.5 of the CASA II sperm analysis software and the manufacturer’s recommended settings. For membrane viability staining, semen was thawed as previously described. 20 μL of semen was gently mixed with 30 μL Easybuffer B and 50 μL of 1 mg/ml Hoechst 33258. Samples were then incubated at 35 °C for 2 minutes, loaded into pre-warmed four chamber slides and imaged using the Animal Motility Viadent package on the same IVOS II system and software.

### Acrosome Integrity

Acrosome integrity measurements were adapted from previously described methods^34^,^35^. Frozen semen straws were thawed in a water bath set at 35 °C for 30 seconds. Semen was then transferred to 1.7 mL microcentrifuge tube and incubated at 35 °C for one hour. 20 μL of semen solution was spread on a microscope slides and air-dried for 15 min. The slides were fixed by immersion in absolute methanol for 15 min. Slides were rinsed in PBS baths twice for 5 min each, transferred to a bath containing 25 μg/mL PNA-FITC for 30 min, and rinsed in three PBS baths for five minutes each. Slides were gently dried using compressed air. One drop of Fluoroshield^TM^ with DAPI Histology Mounting Medium was then applied to each slide and acrosome integrity was measured using fluorescence microscopy as previously described^36^.

### Protoypes

Standard canisters of a YDS-3 Dewar (Chart, Garfield Heights, OH) were used as the platform for prototype construction. The canister for Prototype 1 was modified by welding a circular piece of sheet metal to seal the canister bottom and drilling 3.175 mm diameter holes at the canister midpoint. A custom aluminum ring, 2.1 mm thick, was designed to fit snuggly on the inside of the canister, 5 mm above the holes at the midpoint extending 12 mm downward (Fig. 3b element v). A 1.25 mm gap allowed the LN to flow through the holes at the midpoint.

Prototype 2 was fitted snuggly with a 5 mm thick Cryogel® (Aspen Aerogels, Northborough, MA) blanket against the base and walls. Prototype 3 was fitted an aluminum block with eight 7.2 mm thru holes into a standard canister.

### Instrumented Semen Straw

A bundle of three 36 AWG Type T thermocouples (Omega, Stamford, CT) was inserted into a semen straw. The thermocouple tips were positioned 34 mm, 79 mm, and 124 mm from the crimped end of the straw. The bundle was secured to the crimped end of the straw using 19 mm Kapton tape, waxed lacing cord, and tapered round rubber plugs. Each thermocouple was connected to a data acquisition system with a LabVIEW interface (National Instruments, Austin, TX) and sampled at 1 Hz. All thermocouples were two-point-calibrated using a water bath and LN bath.

### Thermal Cycling Experiments

Thermal cycling experiments were performed in a laboratory with ambient conditions measuring 22 ± 1 °C. A total of six 0.5 ml semen straws occupied the canister for all exposures. For experiments utilizing a full LN Dewar, canisters were returned to the Dewar for at least one minute between exposures. For experiments utilizing a 25% LN Dewar, canisters were returned to the Dewar for at least ten minutes between exposures. Calibration experiments determined that these durations were sufficient to return the semen straw to LN temperatures.

### Statistical Analyses

All data were analyzed in either Prism v6.05 (GraphPad Software Inc., La Jolla, CA) or R v3.1.1^37^. All data were checked for normality with a Shapiro-Wilks normality test prior to analyses. All data met assumptions of normality. Data on sperm motility, viability, and acrosome integrity were analyzed with linear regression. Data on the amount of LN lost during cycling of the canister were analyzed with an Analysis of Variance and, upon significance of the overall test, post-hoc analyses were conducted using a Dunnett’s test (comparing each prototype against just the standard canister, or control). *P*-values for post-hoc comparisons are adjusted for multiple comparisons. Note that data presented in Fig. 2a for intact acrosomes, Fig. 2b for the 1-minute exposures, and Fig. 6a for the standard canister are all the same.

## Additional Information

**How to cite this article**: Lieberman, D. *et al.* Maintaining semen quality by improving cold chain equipment used in cattle artificial insemination. *Sci. Rep.* **6**, 28108; doi: 10.1038/srep28108 (2016).

## Supplementary Material

Supplementary Information
