# Supplementary material for: Maintaining semen quality by improving cold chain equipment used in cattle artificial insemination
Source: Sci Rep. 2016 Jun 17;6:28108. doi: 10.1038/srep28108 (PMC4911568; doi:10.1038/srep28108)
Supplement: Supplementary Information [file srep28108-s1.pdf]

Supplementary Material to: **"Maintaining semen quality by improving cold chain equipment used in cattle artificial insemination"**

Daniel Lieberman[1,]*, Elizabeth McClure[1], Stephen Harston[1], Damian Madan[1,]*

[1] Intellectual Ventures Laboratory, Bellevue, Washington, United States of America.

* To whom correspondence should be addressed: dmadan@intven.com and dalieberman@intven.com

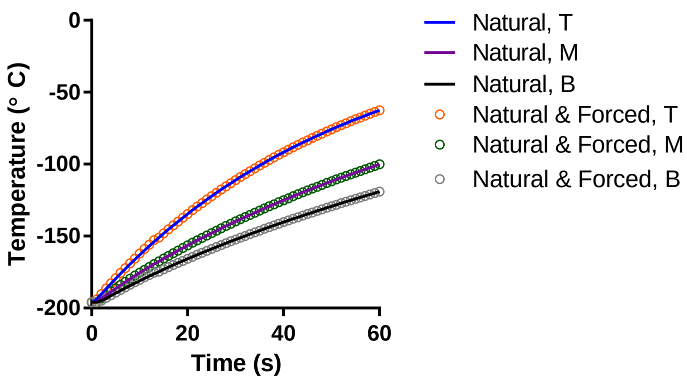

Supplementary Figure 1. Natural convection is largely responsible for the temperature rise inside the canister. Temperature measurements at the top (T), middle (M), and bottom (B) of a semen straw removed from a full 3 L Dewar were simulated using a Multiphysics modeling tool (COMSOL). Simulations were performed with natural convection alone (Natural) or in the presence of forced convection (Natural & Forced) that results when the canister is removed from the Dewar. The degree to which the plots overlay suggests that natural convection is a driving factor and likely marginalizes forced convection effects.

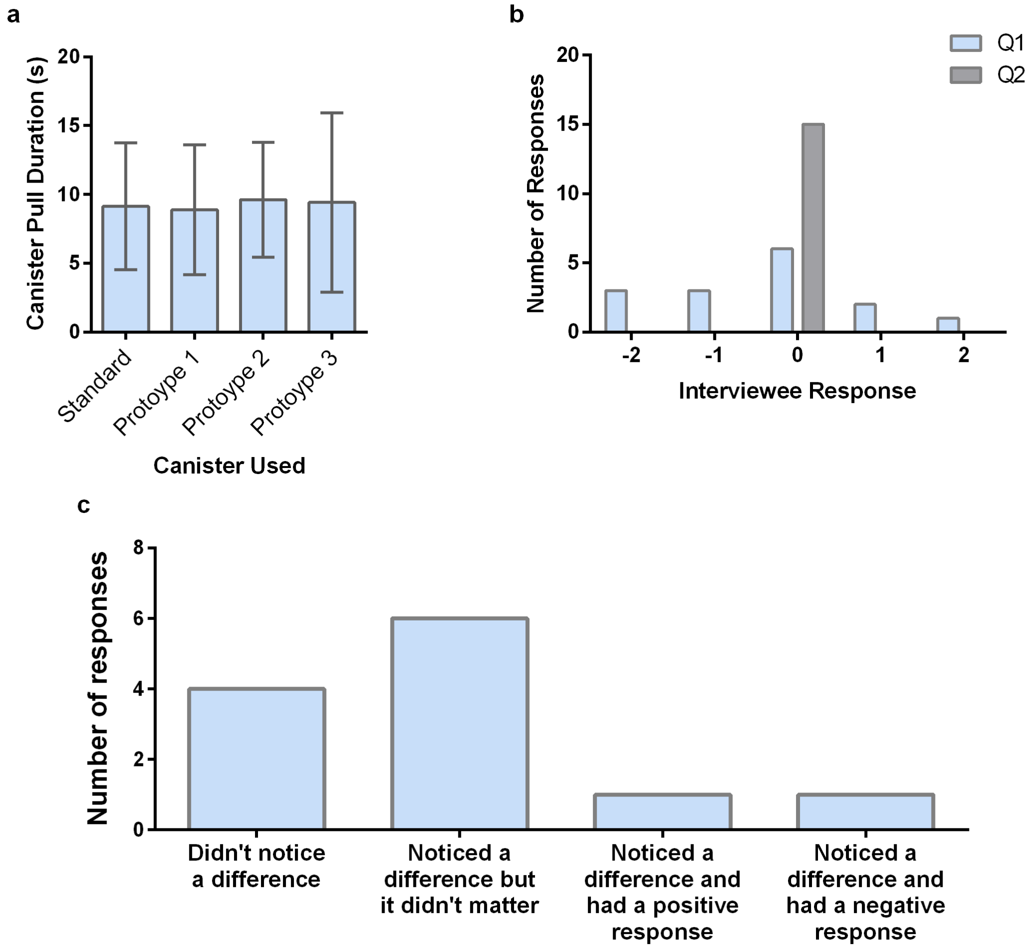

Supplementary Figure 2. Kenyan AI professionals found minimal differences between how the prototypes and standard canisters were handled. (a) The time elapsed while study participants accessed a standard canister (n = 34) or prototypes 1 (n = 31), 2 (n = 36), or 3 (n = 33) are shown. Data were analyzed with ANOVA and were log-transformed for normality prior to analyses. There was no significant difference between any of the four canister types in the time needed to remove the canister from a Dewar ($F_{3,130} = 0.49$, $P = 0.69$). (b) AI professionals responded to two survey questions: "Same or different difficulty removing the canisters from this test compared to your own Dewar?" (Q1) and "Same or different difficulty removing straws from the prototype canisters as compared to the standard canister?" (Q2). Responses (n = 15) were assigned values -2, -1, 0, 1, and 2 corresponding to much easier, a little easier, no difference, a little harder and much harder, respectively. Data for Q1 were analyzed with a one-sample Student's t-test comparing response scores to a null hypothesis of no difference (score of 0) and found no significant difference ($t_{14} = -1.1$, $P = 0.29$). Question 2 responses were all identical, that there was no difference in removing straws from the canister. (c) Responses to the question, "While completing the tasks, did you notice anything different about any of the canisters?" are shown (n = 12). Only one respondent indicated a positive difference and one indicated a negative difference, whereas the other 10 indicated no meaningful difference between the handling of different canisters.

Supplementary Table 1. In a partially filled Dewar, Prototype 2 outperforms the standard canister with and without plastic goblets. Data displayed in Figs. 4D and 4E at 20, 40, and 60 seconds of exposure were compared using separate 1-way ANOVAs for each thermocouple location. Tukey post-hoc comparisons were used to compare the three canister types: standard (SC), prototype 2 (P2) and goblet (G). Reported *P*-values for pairwise comparisons have been adjusted for multiple comparisons.

Time (s)	Thermocouple location	Pairwise comparison	*F*-statistic	*P*-value
20	Top		19.55	0.002
		SC–P2		0.002
		SC–G		0.103
		P2–G		0.023
	Middle		300.5	<0.00001
		SC–P2		< 0.001
		SC–G		< 0.001
		P2–G		0.005
	Bottom		4.62	0.061
		SC–P2		0.065
		SC–G		0.13
		P2–G		0.87
40	Top		26.97	0.001
		SC–P2		< 0.001
		SC–G		0.095
		P2–G		0.008
	Middle		2153.0	<0.00001
		SC–P2		< 0.001
		SC–G		< 0.001
		P2–G		< 0.001
	Bottom		453.6	< 0.00001
		SC–P2		< 0.001
		SC–G		< 0.001
		P2–G		0.001
60	Top		36.78	0.0004
		SC–P2		< 0.001
		SC–G		0.12
		P2–G		0.002
	Middle		3298.0	< 0.00001
		SC–P2		< 0.001
		SC–G		< 0.001
		P2–G		< 0.001
	Bottom		693.5	< 0.00001
		SC–P2		< 0.001
		SC–G		< 0.001
		P2–G		< 0.001

Supplementary Methods

Field Assessment

The field assessment was carried out in Kenya by conducting 17 one-on-one interviews, mock insemination workstations with AI technicians, and 30 interviews in group settings that included seventeen practicing AI technicians, four semen distributers, two senior AI educators and seven leaders, managers and policy directors in the dairy sector in Kenya. This assessment was not a controlled study designed to capture a representative cross section of the inseminator population. All results are qualitative. Participants were introduced to prototypes 1, 2, 3 and standard canisters within full 3L Dewars. Participants were asked to access semen straws from within each canister. Interactions were recorded and the time that to access a semen straw from each canister was recorded. Participants were then asked a series of questions based on their experiences.
